# A Retrospective Analysis of Hand Conditions Treated Using the Wide Awake Local Anesthesia No Tourniquet (WALANT) Technique at a Teaching Hospital

**DOI:** 10.7759/cureus.90198

**Published:** 2025-08-15

**Authors:** Talia Gabay, Yu Fei Jia, Neena de Frey, Tebogo Ramothata, Chikonde Maisela, Antionette Colloty, Nehume Thandie Maseko, Marule Paul Kgagudi

**Affiliations:** 1 Orthopedic Surgery, Wits University, Johannesburg, ZAF

**Keywords:** complications, failure rate, hand pathology, medical costs, walant technique

## Abstract

Background: Musculoskeletal trauma remains on the rise, particularly in low- and middle-income countries (LMICs). Hand injuries are equally prevalent, with patients in LMICs being 5 to 10 times more likely to be affected. Patients who sustain hand injuries are at risk of experiencing temporary or permanent loss of hand function, severely impacting activities of daily living. Early treatment intervention can reduce the risk of long-term disability. The Wide Awake Local Anesthesia No Tourniquet (WALANT) technique, since its development, has revolutionized hand surgery with reported benefits. Our study aimed to analyze the scope of hand pathologies treated under WALANT and its associated failure rate and complications in our context.

Materials and methods: We conducted a retrospective clinical audit of patients treated for hand pathology with WALANT at Charlotte Maxeke Johannesburg Academic Hospital (CMJAH) between 1 July and 31 December 2016. Demographic, pathology, and intraoperative data were collected from the hospital admissions, ward, and theater records of patients who met our inclusion criteria. Procedure waiting times and length of hospital stay were calculated from recorded dates and times. All collected data were de-identified and handled using REDCap (Vanderbilt University, Nashville, TN, USA) and Microsoft Excel version 16.51 (Microsoft Corp., Redmond, WA, USA) for storage and analysis. Descriptive statistics were employed, with measures of central tendency (mean, median, mode) and dispersion (standard deviation) calculated using the Data Analysis ToolPak in Microsoft Excel.

Results: Data for 416 patients were included for analysis after all exclusions. The majority of patients were Black males. Employed individuals and the right hand were affected slightly more than the unemployed and the left hand, respectively. The fingers were affected slightly more than the hand (234 (22.6%); 163 (19.47%)), and infections (152 (36.54%)) were more frequently treated, followed by lacerations (131 (31.49%)). The average procedure waiting time and the length of hospital stay were significantly low, with an equally low complication (4/416 (0.96%)) and failure rate (91/416 (22.12%)).

Conclusions: The practical advantages and clinical safety of WALANT in the South African context provide valuable insights into its role in enhancing healthcare delivery in LMICs. Our study demonstrated a high success rate, minimal complications, and significant cost-effectiveness in a broad scope of hand pathologies treated surgically under WALANT. This study contributes to the growing body of evidence supporting WALANT as a viable solution for managing hand conditions in settings where traditional methods, such as the use of general anesthesia and the availability of a theater, are often limited by financial and logistical constraints.

## Introduction

Musculoskeletal trauma injuries remain a significant health burden, particularly in low- and middle-income countries (LMICs) [1]. Among these, hand injuries are notably prevalent, with patients in LMICs 10 times more likely to suffer from severe and complex hand injuries [2]. Severe hand injuries put one at risk of experiencing temporary or permanent loss of hand function, impacting activities of daily living [3]. Thus, timely and appropriate hand injury treatment could reduce the risk of long-term disability [3]. However, in LMICs like South Africa, significant delays in accessing and receiving hospital care have been identified, exacerbating the burden on healthcare systems and delaying necessary surgical care, which can result in long-term sequelae and disability [4]. This situation highlights the critical need for innovative, resource-efficient surgical techniques to enhance patient outcomes in these resource-limited settings.

The Wide Awake Local Anesthesia No Tourniquet (WALANT) technique, developed by Dr. Lalonde, has become a revolutionary method in hand surgery [5]. Usually, upper limb surgeries are performed under general anesthesia (GA) while using a tourniquet to ensure a bloodless surgical field [6]. WALANT involves the creation of a bloodless surgical field without using a tourniquet, offering efficient and rapid management of trauma and elective hand surgery cases [7]. WALANT utilizes a solution containing a local anesthetic composed of 1% lignocaine and 1:100,000 epinephrine, buffered with sodium bicarbonate in a 1:10 ratio to neutralize the solution’s pH [5,6,8]. Epinephrine acts as a vasoconstrictor, maintaining hemostasis, while lignocaine provides effective local anesthesia [8]. The addition of sodium bicarbonate reduces the acidity of the lignocaine, mitigating the discomfort typically associated with its administration [8]. In the rare event of digital ischemia, phentolamine can be used to reverse epinephrine’s vasoconstrictive effects [6,9].

WALANT eliminates the need for both sedation and an anesthetist, making it particularly valuable in resource-limited settings and for high-risk patients with comorbidities that contraindicate a GA [6,8,10,11]. By keeping the patient awake, the systemic complications associated with GA are avoided, making it a safer alternative for at-risk populations [12,13]. Additional advantages over traditional local anesthesia with a tourniquet include substantially lower injection pain, reduced postoperative pain, decreased reliance on analgesia postoperatively, and higher overall patient satisfaction with the procedure compared to conventional methods [14]. No tourniquet minimizes patient discomfort, allowing for longer procedures without the added tourniquet time limits [12]. An awake patient allows for intraoperative functional assessments when necessary [15]. The above advantages make for an outpatient setting and remote-area applicability and use with a further reduction in costs and improved access to procedures [16]. WALANT also offers flexibility, as the local anesthetic does not require the patient to be sober, a particularly relevant factor in our South African setting, with alcohol significantly associated with trauma [12,17].

WALANT has shown utility in a broad spectrum of hand pathologies, ranging from carpal tunnel syndrome and trigger finger to hand joint replacements [14,18-20]. Beyond the hand, application has expanded into various other surgical domains, widening the scope of use [21-24].

The successful implementation of WALANT in resource-limited settings demonstrates its revolutionary effect with substantial benefits in efficiency and cost reduction. A pertinent example is its adoption at 1 Military Hospital (1MH) in South Africa [25]. That implementation led to a significant reduction in costs, with the institution saving up to R27,962 per procedure [25]. Additionally, WALANT reduced the waiting times for elective surgeries in the South African public health sector [25,26]. At 1MH, the average waiting time for upper limb surgeries reduced from six months to six weeks following the adoption of WALANT, minimizing costs and complications [25].

Therefore, WALANT represents a transformative approach in surgical practices, particularly in resource-limited settings due to financial and logistical constraints [10,11]. The technique enables safer, faster, and cheaper surgeries with an awake patient [10,13]. Despite existing studies on the efficacy and advantages of WALANT, there is limited data on its complications and failure rates, as well as its effectiveness across a diverse range of pathologies [14]. Understanding these factors in the South African context could provide essential insights into the broader implications of WALANT for improving patient outcomes in LMICs. Our research aims to address gaps and contribute to evidence supporting WALANT as a viable solution for managing musculoskeletal trauma, enhancing healthcare service delivery, and reducing surgical backlogs.

## Materials and methods

We conducted a retrospective clinical audit of patients treated for hand pathology with WALANT at Charlotte Maxeke Johannesburg Academic Hospital (CMJAH) between 1 July and 31 December 2016. Demographic, pathology, and intraoperative data were collected from the hospital admissions, ward, and theater records. Additionally, date of admission and length of hospital stay, date of discharge, conversion to GA, as well as any postoperative complications were documented. All collected data were de-identified and handled with REDCap (Vanderbilt University, Nashville, TN, USA) and Microsoft Excel version 16.51 (Microsoft Corp., Redmond, WA, USA) for storage and analysis. The hospital length of stay was calculated by subtracting the admission date from the discharge date. The failure of a WALANT procedure was defined as either the need to convert to GA or the application of a tourniquet within the first five minutes of surgery. Variables were classified as either categorical or continuous. Descriptive statistics were employed, with measures of central tendency (mean, median, mode) and dispersion (standard deviation) calculated using the Data Analysis ToolPak in Microsoft Excel. Data of all patients aged 18 years or older treated for hand pathologies using WALANT were included in the study. Incomplete patient records, treatment under GA, and those who had a tourniquet applied from the surgical start of the procedure were excluded. The Wits Human Research Ethics Committee approved our study with the number M2309049.

## Results

**Figure 1:**
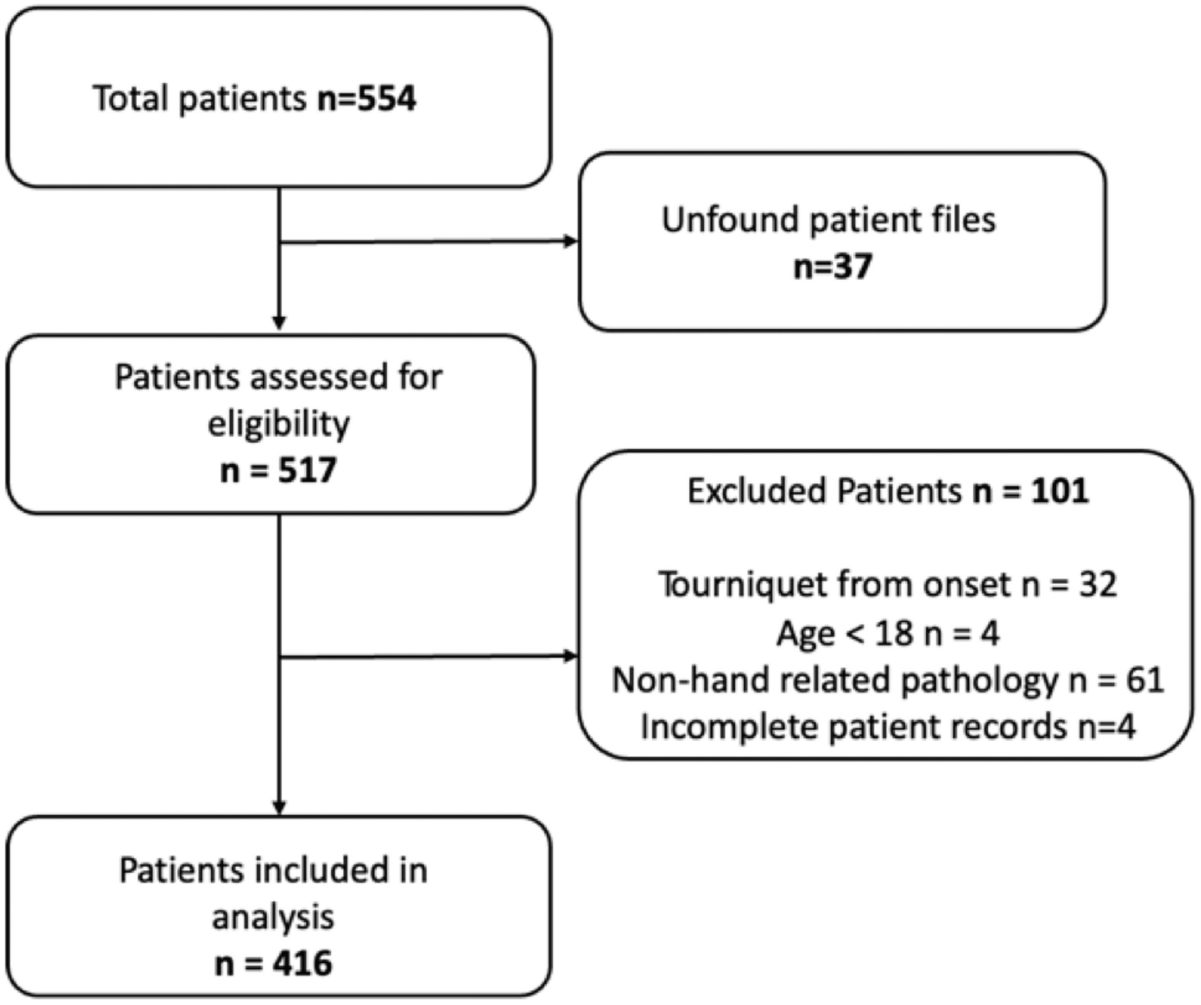
Flowchart of patient file selection process for WALANT hand surgeries at CMJAH This flowchart illustrates the selection process of patient files for inclusion in the study. WALANT: Wide Awake Local Anesthesia No Tourniquet, CMJAH: Charlotte Maxeke Johannesburg Academic Hospital

Records for 416 patients were available for analysis after all exclusions. Figure 1 below illustrates the initial screening process. As seen in Table 1, the majority of patients were Black male patients. The age, employment status, and handedness are tabulated. Table 2 shows the anatomical regional involvement by pathology. The distribution of pathology type treated with WALANT is detailed in Table 3. Infections represented the most prevalent pathology, followed by lacerations and fractures in that order. Detailed pathological sub-categorizations are also tabulated under each group.

**Table 1:** Demographic profile of patients undergoing WALANT hand surgeries at CMJAH Summary of patient demographics, including sex, race, age, occupation status, and handedness for our cohort of patients. The age range of the cohort was 18 to 89 years, with a mean age of 35.72 ± 12.48 years. n=416. WALANT: Wide Awake Local Anesthesia No Tourniquet, CMJAH: Charlotte Maxeke Johannesburg Academic Hospital

Individual-level variables	n	Percentage	Mean ± SD
Sex (%)			
Male	321	77.16	
Female	95	22.84	
Race			
Black	381	91.59	
White	22	5.29	
Colored	7	1.68	
Asian	4	0.96	
Indian	2	0.48	
Occupation status			
Employed	191	45.91	
Unemployed	185	44.47	
Unknown	22	5.29	
Pensioner	18	4.33	
Handedness			
Unknown	215	51.68	
Right	187	44.95	
Left	14	3.37	

**Table 2:** Distribution of hand pathologies treated with WALANT by anatomical location The various anatomical locations were divided into pathologies involving the finger, hand, wrist, multiple zones on the hands, and bilateral hand involvement. Percentages were calculated by using the total number of cases included in our study (n=416). Cases with combined right and left hand involvement. n=4, 0.96%. WALANT: Wide Awake Local Anesthesia No Tourniquet

Location	Right		Left	
	n	Percentage	n	Percentage
Finger	104	25	94	22.6
Hand	81	19.71	82	19.47
Multiple zones	16	3.85	11	4.33
Wrist	11	2.64	6	1.44

**Table 3:** Scope of hand pathologies treated with WALANT Percentages are calculated by using the total number of included patients in the study (n=416). WALANT: Wide Awake Local Anesthesia No Tourniquet

Pathology	Total n	n	Percentage
Infection	152		36.54
Abscess		135	32.45
Sepsis		13	3.13
Paronychia		2	0.48
Gangrene		1	0.24
Cellulitis		1	0.24
Laceration	131		31.49
Unspecified		64	15.38
Tendon		59	14.18
Nerve		6	1.44
Muscle		2	0.48
Fracture	60		14.42
Phalanx		35	8.41
Metacarpal		13	3.13
Unspecified		7	1.68
Scaphoid		3	0.72
Radioulnar		1	0.24
Radial		1	0.24
Traumatic amputation	21		5.05
Nail bed injury	11		2.64
Masses/cysts	11		2.64
Ganglion cyst		3	0.72
Squamous cell carcinoma		3	0.72
Unspecified mass		2	0.48
Granuloma		1	0.24
Basal cell carcinoma		1	0.24
Malignant melanoma		1	0.24
Degloving injury	9		2.16
Foreign body	6		1.44
Burns	5		1.2
Dislocation	3		0.72
Metacarpal		2	0.48
Scaphoid		1	0.24
Necrosis	2		0.48
Other	5		1.2
Contracture		1	0.24
Crush injury		1	0.24
Gangrene		1	0.24
Human bite		1	0.24
Osteoarthritis		1	0.24
Total		416	100

Duration of waiting times from admission to procedure

**Figure 2:**
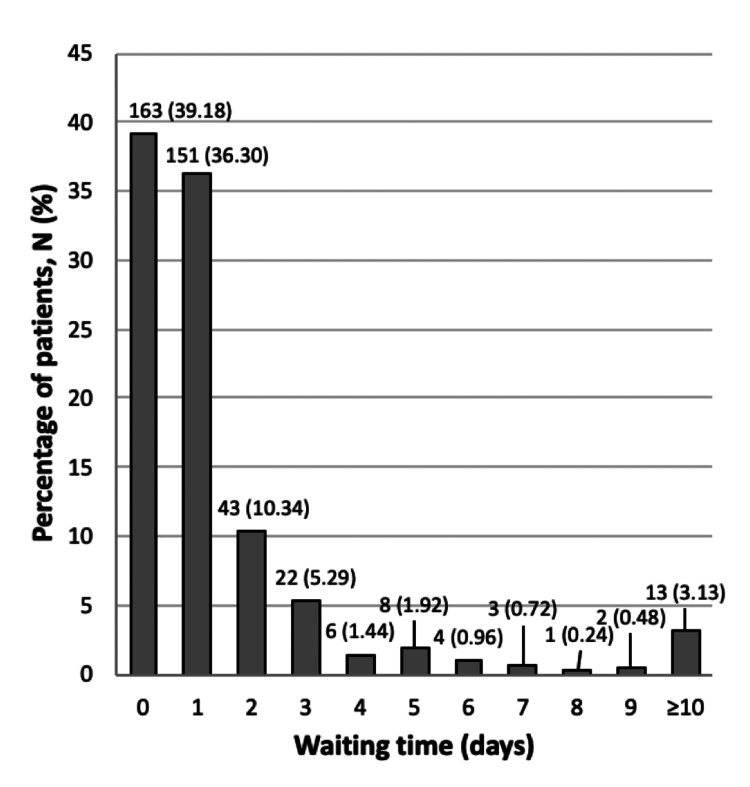
Waiting time duration from admission to WALANT procedure This figure illustrates the waiting times from admission to WALANT for patients treated at CMJAH between 1 July and 31 December 2016. The mean ± SD was 1.75 ± 4.63. The x-axis represents the number of days waited, while the y-axis indicates the percentage of patients corresponding to each waiting time interval. The data highlight the variability in waiting times, with the majority of patients experiencing minimal delays, typically waiting 0 to 1 day before their procedure. SD: standard deviation, WALANT: Wide Awake Local Anesthesia No Tourniquet, CMJAH: Charlotte Maxeke Johannesburg Academic Hospital

The time to procedure varied from 0 to 47 days, with a mean of 1.75 ± 4.36 days. Figure 2 illustrates that 39.18% of patients had their procedure on the same day as admission.

Procedure duration with WALANT

**Figure 3:**
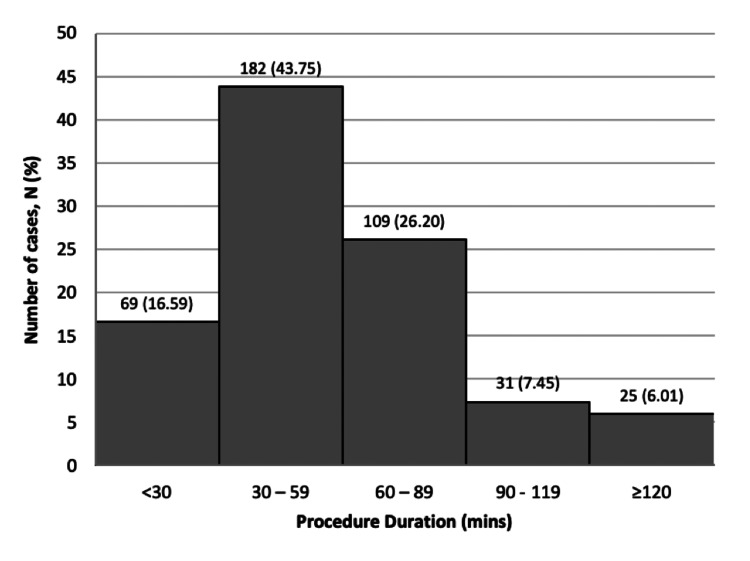
Duration times of hand procedures treated with WALANT The figure represents the total procedure duration in minutes for 416 patients who underwent WALANT at CMJAH. The minimum procedure duration was five minutes, and the longest duration was 225 minutes. The mean ± SD was 54.58 ± 32.43 minutes, with the majority of procedures occurring in under 60 minutes (60.34%). Percentages are calculated by using the total number of included patients in the study (n=416). SD: standard deviation, WALANT: Wide Awake Local Anesthesia No Tourniquet, CMJAH: Charlotte Maxeke Johannesburg Academic Hospital

Figure 3 illustrates the procedure durations while using WALANT, which ranged from five to 225 minutes, with a mean of 54.58 ± 32.43 minutes. Notably, 60.34% of procedures were completed within an hour. Nearly half (43.75%) of the cases took between 30 and 59 minutes.

Average length of hospital stay

**Figure 4:**
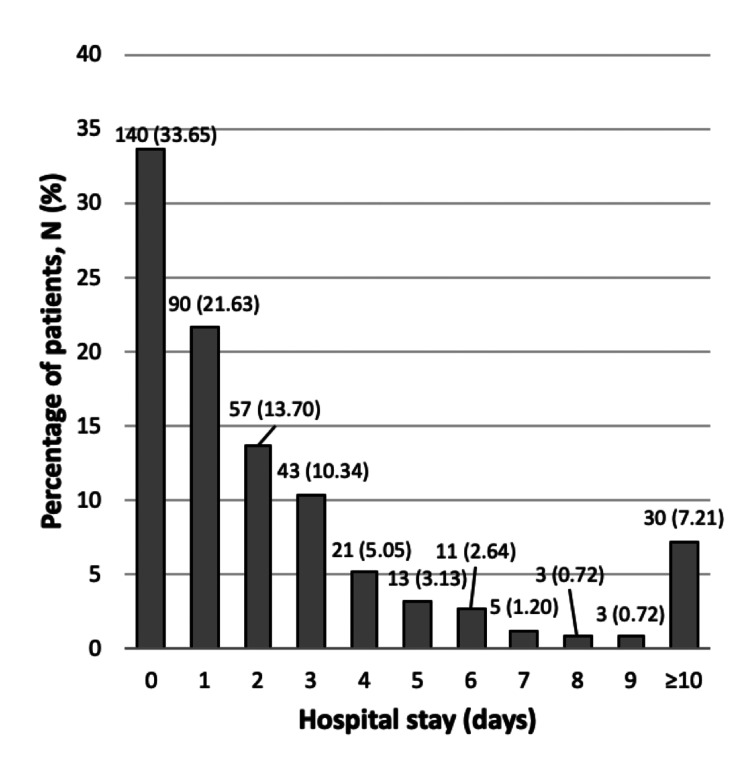
Length of hospital stay associated with WALANT procedures This figure presents the distribution of hospital stay durations for 416 patients who underwent hand surgery using WALANT at CMJAH. The data reveal a range of stay lengths, with the majority of patients discharged within 0 to 1 day. The mean ± SD duration of hospital stay was 3.08 ± 6.30 days. Each duration category's percentages are also indicated, offering valuable insight into the typical recovery timelines associated with these surgical procedures. SD: standard deviation, WALANT: Wide Awake Local Anesthesia No Tourniquet, CMJAH: Charlotte Maxeke Johannesburg Academic Hospital

The average hospital stay was 3.08 ± 6.30 days, with most patients (33.65%) being discharged on the same day as their procedure and 21.63% on the first postoperative day. Over half (55.28%) of patients were discharged within 24 hours of their procedure. These findings are depicted in Figure 4.

Complications included sepsis (0.72%) and necrosis (0.24%). No complications were reported in 99.04% of the cohort. A total of 90 cases had a tourniquet applied during the procedure, and only one case was converted to GA, equating to a failure rate of 22.12% and therefore a WALANT success rate of 77.88% (Table 4).

**Table 4:** Failure rate and complications detected during WALANT The total number of tourniquet applications was calculated to determine the failure rate after five minutes or more after the start of the procedure or the conversion to general anesthesia. This is represented as a percentage by using the total number of included patients (n=416). WALANT: Wide Awake Local Anesthesia No Tourniquet

Individual-level variables	n	Percentage	Total (%)
Complications			0.96
Sepsis	3	0.72	
Necrosis	1	0.24	
Failure rate			22.12
Tourniquet application ≥ 5 min after start of WALANT	90	21.88	
General anesthesia	1	0.24	

## Discussion

Our clinical audit showed the impact and applicability of WALANT in an LMIC setting, where a broad scope of hand pathologies was treated with WALANT. The study also reflected the high volume of cases (n=416) performed over six months in a resource-limited environment, highlighting the procedure's feasibility and applicability in such settings. The application of WALANT in trauma-related pathologies, such as lacerations, fractures, and amputations, as well as in non-trauma conditions like masses/cysts and infections, was well-represented in our study, akin to published data [8,12,27,28]. Additionally, the use of WALANT for De Quervain's tenosynovitis, trigger finger, carpal tunnel syndrome, and ganglion cysts was well-described by de Buys et al. at Helen Joseph Hospital, South Africa [8]. This highlights the versatility of WALANT for a variety of hand conditions. Infections are not frequently documented in the literature as typical indications for WALANT. Interestingly, some regard infections as a contraindication for WALANT, arguing that the acidic environment in infected tissues reduces the efficacy of the local anesthetic [13]. However, newer research, including findings from Degreef and Lalonde, suggests that infections are no longer contraindications for WALANT [12]. This is a significant finding given that infections are prevalent in LMICs. In our study, 36.54% of cases were infected and adequately treated under WALANT.

Studies by LeBlanc et al. and Chatterjee et al. [29,30] demonstrated that WALANT procedures for carpal tunnel release and trigger finger release could be performed in half the time of similar surgeries under GA. In our study, the average procedure duration was 54.58 minutes, with 60.34% of cases completed under 60 minutes. This aligns with findings from Seretis et al., who reported an average procedure duration of 56 minutes, while a systematic review by Gouveia et al. noted an average of 60.4 minutes [6,31]. However, our procedure times are slightly longer than those reported in other studies, such as Mohammed et al. (34.62 minutes), Sasor et al. (21.45 minutes), and Srisai and Jianmongkol (36.80 minutes) [32-34]. This variation may be attributed to differences in surgeon experience, inclusion criteria, and/or procedural complexity.

Additionally, the fact that our surgeries were conducted in an operating theater rather than an outpatient procedure setting may have contributed to the longer times due to porters and additional administrative delays. Despite this, our procedure duration remains significantly shorter than that of those requiring GA, which often exceeds two hours [35]. The latter highlights the potential of WALANT in reducing surgical backlogs when implemented effectively [25].

In our cohort, 55.28% of patients were discharged on day one of admission to the hospital. This contrasts with studies reporting hospital stays of over two days for hand surgeries under GA, potentially cutting hospital-related costs by 25-50% [28]. These faster discharges are particularly beneficial in resource-limited settings like South Africa, where hospital efficiency is critical. Our findings highlight WALANT’s potential to enhance care delivery in LMICs by shortening hospital stays and easing the strain on healthcare resources. Furthermore, existing literature also shows shortened preoperative waiting times, a trend supported by our data, where 75.48% of patients waited for one day or less for their procedure [25]. This timely intervention is vital in hand pathologies to prevent functional loss and minimize complications [3].

The cost benefits of WALANT can be inferred from the results. Firstly, the anesthetic requirements used in WALANT are less expensive compared to those of GA [25]. Secondly, WALANT eliminates the need for an anesthetist, reducing personnel costs [6]. Thirdly, the majority (55.28%) of patients in our study required hospital stays of less than 24 hours. Fourthly, the shorter duration of WALANT procedures allows for more operations to be completed within the same time frame as those performed under GA [35]. Fifthly, this reduction in operating time decreases hospital backlogs and prevents unnecessary surgical delays [31]. Additionally, WALANT’s ability to provide real-time functional assessment during surgery may reduce the need for future re-operations, contributing to further cost savings [15]. And finally, WALANT uses fewer resources, as smaller sterile fields are required [8].

A feared complication of the use of epinephrine, a vasoconstrictor, in anesthetic block solutions of the hand is the perceived high risk of digital ischemia. However, studies show this minimal risk, especially when combined with lidocaine [13,28]. In our study, no cases of digital ischemia or mortality were reported; this aligns with the current literature, further reinforcing the safety of WALANT [8]. Our study demonstrated an overall low complication rate of 0.96%, which included wound sepsis (0.72%) and skin necrosis (0.24%). This complication rate is consistent with previous findings [36-38]. Importantly, these complications developed in the postoperative period. For instance, one 70-year-old patient developed sepsis after a 24-day hospital stay, of which 17 days were spent awaiting surgery for a radio-ulnar fracture with another hospital department. This case suggests that longer waiting times or extended hospital stays may increase the risk of septic complications. Nonetheless, a meta-analysis by Evangelista et al. found no significant difference in complication rates between WALANT and the conventional combination of tourniquet and GA, further supporting the safety of this technique [39].

The failure rate for WALANT is a critical finding in our study, as there is very limited published data on this aspect. In our analysis of 416 total cases, the failure rate of WALANT was found to be 22.12%. Of this, 0.24% required conversion to GA due to anxiety, with 21.88% of those patients requiring a tourniquet because adequate hemostasis could not be achieved during the procedure. Although these cases are classified as failures according to the definition of WALANT of local anesthesia without a tourniquet, it is essential to emphasize that patient safety is not compromised in these situations, as the use of a tourniquet is still considered a standard operative practice [6]. Even when classified as failures, the procedures were completed, ensuring that patient care continued without interruption. While WALANT presents an opportunity for more comfortable surgeries, it is important to note that tourniquets can still be used in awake patients. Studies indicate that patients can tolerate a tourniquet for up to 25 minutes on the forearm and around 18 minutes on the arm due to pain intolerance [28]. Moreover, the intraoperative bleeding observed in the study was likely due to insufficient waiting times for maximum vasoconstriction based on relatively shorter procedure times. McKee et al. recommend waiting between 27 and 30 minutes post-injection for optimal vasoconstrictive effects, as opposed to the traditional seven minutes [40]. Adhering to these recommendations can minimize intraoperative bleeding, further enhancing the safety of WALANT procedures.

Our study was limited by its retrospective nature, with poor record-keeping and documentation leading to missing information and record exclusions. Also, focusing on a single hospital in South Africa, the findings may not be generalizable to other institutions or regions due to clinician differences. Moreover, the study was only able to capture waiting times from admission, omitting the duration patients spent waiting at home before their procedures. A structured prospective study could give stronger conclusions and recommendations.

## Conclusions

Practical advantages and clinical safety of WALANT in the South African context provide valuable insights into its role in enhancing healthcare delivery in LMICs. Our study demonstrates a high success rate, minimal complications, and significant cost-effectiveness in a wide scope of hand pathologies treated surgically under WALANT. This study contributes to the growing body of evidence supporting WALANT as a viable solution for managing hand conditions in settings where traditional methods, such as the use of GA and the availability of a theater, are often limited by financial and logistical constraints.

## References

[1] Crowe CS, Massenburg BB, Morrison SD (2020). Global trends of hand and wrist trauma: a systematic analysis of fracture and digit amputation using the Global Burden of Disease 2017 Study. Inj Prev.

[2] Sayyari Y, Kardar MH, Sadeghian F, Mirrezaie SM (2022). The impact of socioeconomic status on hand injury severity. Hand Surg Rehabil.

[3] Dębski T, Noszczyk BH (2021). Epidemiology of complex hand injuries treated in the plastic surgery department of a tertiary referral hospital in Warsaw. Eur J Trauma Emerg Surg.

[4] Mac Quene T, Smith L, Odland ML, Levine S, D'Ambruoso L, Davies J, Chu K (2022). Prioritising and mapping barriers to achieve equitable surgical care in South Africa: a multi-disciplinary stakeholder workshop. Glob Health Action.

[5] Lalonde D, Bell M, Benoit P, Sparkes G, Denkler K, Chang P (2005). A multicenter prospective study of 3,110 consecutive cases of elective epinephrine use in the fingers and hand: the Dalhousie Project clinical phase. J Hand Surg Am.

[6] Seretis K, Boptsi A, Boptsi E, Lykoudis EG (2023). The efficacy of wide-awake local anesthesia no tourniquet (WALANT) in common plastic surgery operations performed on the upper limbs: a case-control study. Life (Basel).

[7] Choukairi F, Ibrahim I, N A Murphy R, Reid AJ, Winterton RI, Bedford JD, Wong JK (2021). Development of the Manchester wide-awake hand trauma service in 2020: the patient experience. J Hand Surg Eur Vol.

[8] de Buys M, Tsama M, Aden AA (2022). Patient satisfaction following wide awake local anaesthetic no tourniquet hand surgery. SA Orthop J.

[9] McCaughran PW, Zargaran D, Southall C, Kokkinos C, Caine P, Nikkhah D, Mosahebi A (2022). WALANT: Perceptions, approaches, and contraindications in a tertiary hand surgery unit. Hand Surg Rehabil.

[10] Shahid S, Saghir N, Saghir R, Young-Sing Q, Miranda BH (2022). WALANT: a dscussion of indications, impact, and educational requirements. Arch Plast Surg.

[11] Connors KM, Kurtzman JS, Koehler SM (2023). Successful use of WALANT in local and regional soft tissue flaps: a case series. Plast Reconstr Surg Glob Open.

[12] Degreef I, Lalonde DH (2024). WALANT surgery of the hand: state of the art. EFORT Open Rev.

[13] Fish MJ, Bamberger HB (2025). Wide-awake local Anesthesia no tourniquet (WALANT) hand surgery. StatPearls [Internet].

[14] Ki Lee S, Gul Kim S, Sik Choy W (2020). A randomized controlled trial of minor hand surgeries comparing wide awake local anesthesia no tourniquet and local anesthesia with tourniquet. Orthop Traumatol Surg Res.

[15] Maliha SG, Cohen O, Jacoby A, Sharma S (2019). A cost and efficiency analysis of the Walant technique for the management of trigger finger in a procedure room of a major city hospital. Plast Reconstr Surg Glob Open.

[16] Zargaran D, Zargaran A, Nikkhah D, Mosahebi A (2021). WALANT protocol: stop before you block. J Plast Reconstr Aesthet Surg.

[17] Foster M, Du Plessis J, Van Vuuren MJ, Jingo M, Pietrzak R (2022). The impact of the COVID-19 lockdown restrictions on orthopaedic trauma admissions in a central academic hospital in Johannesburg. SA Orthop J.

[18] Far-Riera AM, Perez-Uribarri C, Serrano MJ, González JM (2023). Impact of Walant hand surgery in a secondary care hospital in Spain. Benefits to the patient and the health system. J Hand Surg Glob Online.

[19] Gunasagaran J, Sean ES, Shivdas S, Amir S, Ahmad TS (2017). Perceived comfort during minor hand surgeries with wide awake local anaesthesia no tourniquet (WALANT) versus local anaesthesia (LA)/tourniquet. J Orthop Surg (Hong Kong).

[20] Müller CT, Christen T, Heidekruger PI (2018). Wide-awake anesthesia no tourniquet trapeziometacarpal joint prosthesis implantation. Plast Reconstr Surg Glob Open.

[21] Ahmad AA, Ubaidah Mustapa Kamal MA, Ruslan SR, Abdullah S, Ahmad AR (2020). Plating of clavicle fracture using the wide-awake technique. J Shoulder Elbow Surg.

[22] Bilgetekin YG, Kuzucu Y, Öztürk A, Yüksel S, Atilla HA, Ersan Ö (2021). The use of the wide-awake local anesthesia no tourniquet technique in foot and ankle injuries. Foot Ankle Surg.

[23] Gueffier X, Gueffier E, Lalonde D (2022). Mini-open one-stage flexor digitorum profundus tendon graft for jersey finger under WALANT with ultrasound assistance: a case report of a new technique. Hand Surg Rehabil.

[24] Kurtzman JS, Etcheson JI, Koehler SM (2021). Wide-awake local anesthesia with no tourniquet: an updated review. Plast Reconstr Surg Glob Open.

[25] Koch O (2022). How to start WALANT practice in South Africa: “service with a smile if you are willing to wait awhile.”. J Hand Surg Glob Online.

[26] Valente R, Testi A, Tanfani E (2009). A model to prioritize access to elective surgery on the basis of clinical urgency and waiting time. BMC Health Serv Res.

[27] Poggetti A, Nucci AM, Giesen T, Calcagni M, Marchetti S, Lisanti M (2018). Percutaneous intramedullary headless screw fixation and wide-awake anesthesia to treat metacarpal fractures: early results in 25 patients. J Hand Microsurg.

[28] Sawhney A, Thacoor A, Nagra R, Geoghegan L, Akhavani M (2024). Wide Awake local anesthetic no tourniquet in hand and wrist surgery: current concepts, indications, and considerations. Plast Reconstr Surg Glob Open.

[29] Leblanc MR, Lalonde J, Lalonde DH (2007). A detailed cost and efficiency analysis of performing carpal tunnel surgery in the main operating room versus the ambulatory setting in Canada. Hand (N Y).

[30] Chatterjee A, McCarthy JE, Montagne SA, Leong K, Kerrigan CL (2011). A cost, profit, and efficiency analysis of performing carpal tunnel surgery in the operating room versus the clinic setting in the United States. Ann Plast Surg.

[31] Gouveia K, Harbour E, Gazendam A, Bhandari M (2024). Fixation of distal radius fractures under wide-awake local anesthesia: a systematic review. Hand (N Y).

[32] Mohammed O, Attia J, Abdelzaher T (2022). WALANT technique versus conscious sedation in minor hand surgery. Minia J Med Res.

[33] Sasor SE, Cook JA, Duquette SP (2020). Tourniquet use in wide-awake carpal tunnel release. Hand (N Y).

[34] Srisai C, Jianmongkol S (2022). Comparison of effectiveness in flexor tendon hand repairs between WALANT technique and conventional method. J Southeast Asian Orthop.

[35] Bismil M, Bismil Q, Harding D, Harris P, Lamyman E, Sansby L (2012). Transition to total one-stop wide-awake hand surgery service-audit: a retrospective review. JRSM Short Rep.

[36] Lawand J, Hantouly A, Bouri F, Muneer M, Farooq A, Hagert E (2024). Complications and side effects of wide-awake local anaesthesia no tourniquet (WALANT) in upper limb surgery: a systematic review and meta-analysis. Int Orthop.

[37] Leblanc MR, Lalonde DH, Thoma A (2011). Is main operating room sterility really necessary in carpal tunnel surgery? A multicenter prospective study of minor procedure room field sterility surgery. Hand (N Y).

[38] Naude JJ, Koch O, Schmidt LW, Le Roux TLB (2021). Positive patient experience of wide wake local anaesthesia no tourniquet (WALANT) hand surgery in the government setting: a prospective descriptive study. SA Orthop J.

[39] Evangelista TM, Pua JH, Evangelista-Huber MT (2019). Wide-awake local anesthesia no tourniquet (WALANT) versus local or intravenous regional anesthesia with tourniquet in atraumatic hand cases in orthopedics: a systematic review and meta-analysis. J Hand Surg Asian Pac Vol.

[40] Mckee DE, Lalonde DH, Thoma A, Dickson L (2015). Achieving the optimal epinephrine effect in wide awake hand surgery using local anesthesia without a tourniquet. Hand (N Y).

